# Twenty-Two Years of Warming, Fertilisation and Shading of Subarctic Heath Shrubs Promote Secondary Growth and Plasticity but Not Primary Growth

**DOI:** 10.1371/journal.pone.0034842

**Published:** 2012-04-12

**Authors:** Matteo Campioli, Niki Leblans, Anders Michelsen

**Affiliations:** 1 Department of Biology, University of Antwerp, Wilrijk, Belgium; 2 Department of Biology, University of Copenhagen, Copenhagen, Denmark; 3 Center for Permafrost (CENPERM), University of Copenhagen, Copenhagen, Denmark; Norwegian University of Science and Technology, Norway

## Abstract

Most manipulation experiments simulating global change in tundra were short-term or did not measure plant growth directly. Here, we assessed the growth of three shrubs (*Cassiope tetragona*, *Empetrum hermaphroditum* and *Betula nana*) at a subarctic heath in Abisko (Northern Sweden) after 22 years of warming (passive greenhouses), fertilisation (nutrients addition) and shading (hessian fabric), and compare this to observations from the first decade of treatment. We assessed the growth rate of current-year leaves and apical stem (primary growth) and cambial growth (secondary growth), and integrated growth rates with morphological measurements and species coverage. Primary- and total growth of *Cassiope* and *Empetrum* were unaffected by manipulations, whereas growth was substantially reduced under fertilisation and shading (but not warming) for *Betula*. Overall, shrub height and length tended to increase under fertilisation and warming, whereas branching increased mostly in shaded *Cassiope*. Morphological changes were coupled to increased secondary growth under fertilisation. The species coverage showed a remarkable increase in graminoids in fertilised plots. Shrub response to fertilisation was positive in the short-term but changed over time, likely because of an increased competition with graminoids. More erected postures and large, canopies (requiring enhanced secondary growth for stem reinforcement) likely compensated for the increased light competition in *Empetrum* and *Cassiope* but did not avoid growth reduction in the shade intolerant *Betula*. The impact of warming and shading on shrub growth was more conservative. The lack of growth enhancement under warming suggests the absence of long-term acclimation for processes limiting biomass production. The lack of negative effects of shading on *Cassiope* was linked to morphological changes increasing the photosynthetic surface. Overall, tundra shrubs showed developmental plasticity over the longer term. However, such plasticity was associated clearly with growth rate trends only in fertilised plots.

## Introduction

The Arctic is the region which will likely experience the most pronounced alteration in climate and environment due to global change [1]. As arctic ecosystems are very sensitive to changes in environmental conditions and store a significant amount (12%) of the global soil carbon (C), extensive research efforts have been made in the last three decades to understand the future feedback of arctic ecosystems to the greenhouse effect and global climate [1]–[3]. In particular, manipulation experiments have been set up to mimic the expected changes in arctic climate and their impact on ecosystems [4]–[6]. Many experiments have focused on the effect of warming during the growing season, of crucial importance for the arctic plant communities adapted to a short and cool summer [7]. Focus has been on plant growth, which (i) can be considered as a surrogate for plant fitness and as such a crucial process for plant subsistence and development, (ii) represents the amount of C taken up annually by the vegetation, and (iii) determines, through the process of C allocation to plant organs with different life-spans and decomposition rates, the C release by the ecosystem in the long-term [8].

The impact of warming on plant growth can be direct or indirect. The direct effect of warming has been mimicked by enhancing air and soil temperature, e.g. with open top chambers [9], [10]. Indirect effects of warming are manifold [11], [12]. However, the increase in nutrient availability through enhanced net mineralization is thought to be one of the most important indirect effects of warming for arctic plant communities, which are commonly nutrient limited [13], [14]. This indirect effect of warming has been mimicked by adding fertilisers during the growing season [15], under ambient or enhanced temperature. A second indirect effect (particularly important in the Subarctic and Low Arctic), is the potential increase in competition due to tree-line advancement and shrub expansion [16]–[18]. Such impact has been mimicked by shading [9], [10]. In the Subarctic and Low Arctic, manipulative experiments have shown that fertilisation has a strong effect on the growth of deciduous species in tussock tundra, and of all vascular species (and particularly graminoids) in heath tundra, whereas warming and shading have small or non significant effects [5], [11]. However, these findings rely mainly on studies not longer than a decade.

In this study, we aimed to broaden the current knowledge on the long-term impact of warming, fertilisation and shading in subarctic ecosystems by assessing the long-term responses in growth of the widespread dwarf-shrubs *Cassiope tetragona* (L.) D. Don., *Empetrum hermaphroditum* Hagerup and the low shrub *Betula nana* L., at a tree-line heath in Northern Sweden after 22 years of manipulation. The experiment is unique as we are not aware of similar well-replicated experiments of such duration in the Subarctic and Low Arctic. Furthermore, the experimental site is particularly suited for this analysis as it was intensively investigated in the first decade of manipulation, providing reports on the shorter term responses of shrub growth to manipulations. In the first decade of manipulation, warming yielded a modest positive response in the growth of *Cassiope* and no response in *Empetrum* and *Betula*, fertilisation led to a positive response in *Cassiope* and particularly in *Empetrum* but not in *Betula*, whereas shading gave a negative response, strong for *Betula* and modest for *Cassiope* and *Empetrum* [5], [13], [19], [20], [21]. After more than two decades of treatment, we expected the growth responses of arctic shrubs to differ from the short-term responses for three reasons. First, the steady changes in community composition, favouring graminoids, which were observed in fertilized plots in the short-term [22], and the competition for light that graminoids exert on prostrate shrubs [5], are likely to negatively affect the shrub growth over the longer term. Second, the mechanisms that buffered the negative effect of shading in the first years of treatment (e.g. usage of stored resources, short-term acclimations) [19], [21], were expected to weaken over the longer term, in particular for the less shade tolerant species such as *Betula* and *Cassiope*. Third, the rate of physiological processes that counterbalanced the positive effect of warming on gross photosynthesis over the shorter term (e.g. respiration, tissue turnover) [9], [11] was expected to decrease because of long-term acclimation [9], [23].

The stem secondary growth of shrubs (cambial growth or increase in stem diameter) accounts for a significant portion of aboveground net primary production in tundra ecosystems (e.g. up to ∼50% at species level (*Salix pulchra* [24]) and ∼20% at plant community level (subarctic heath [25])) and it is sensitive to environmental perturbations [26]. Nevertheless, secondary growth is seldom investigated. The physiological function of secondary growth differs from the one of primary growth. In fact, whereas primary growth assures light interception and photosynthetic uptake, secondary growth sustains the C uptake (e.g. by producing new conduits for water and sugar transport) but also provides the essential mechanical support to the canopy [26]. In a recent study on the growth of arctic shrubs, Campioli et al. [27] found that changes in primary and secondary growth between sites with different environmental conditions were not proportional.

In detail, we tested two hypotheses. Hypothesis 1. The growth responses of arctic shrubs to long-term environmental manipulations differ from the short-term growth responses. Over the longer term, warming was expected to have a more positive effect, fertilisation a less positive effect (or even a negative effect) and shading a more negative effect. Hypothesis 2. The responses of primary and secondary growth to environmental manipulations differ according to the concurrent morphological changes. If the manipulations promoted morphological changes implying enhanced mechanical support for the shrub stem (e.g. increase in shrub height or branching), the response of the secondary growth is expected to differ from the response of the primary growth. On the other hand, if the morphology of the shrub was not altered by the manipulations, primary and secondary growth are expected to present similar response patterns.

## Results

### Growth rates

**Figure 1:**
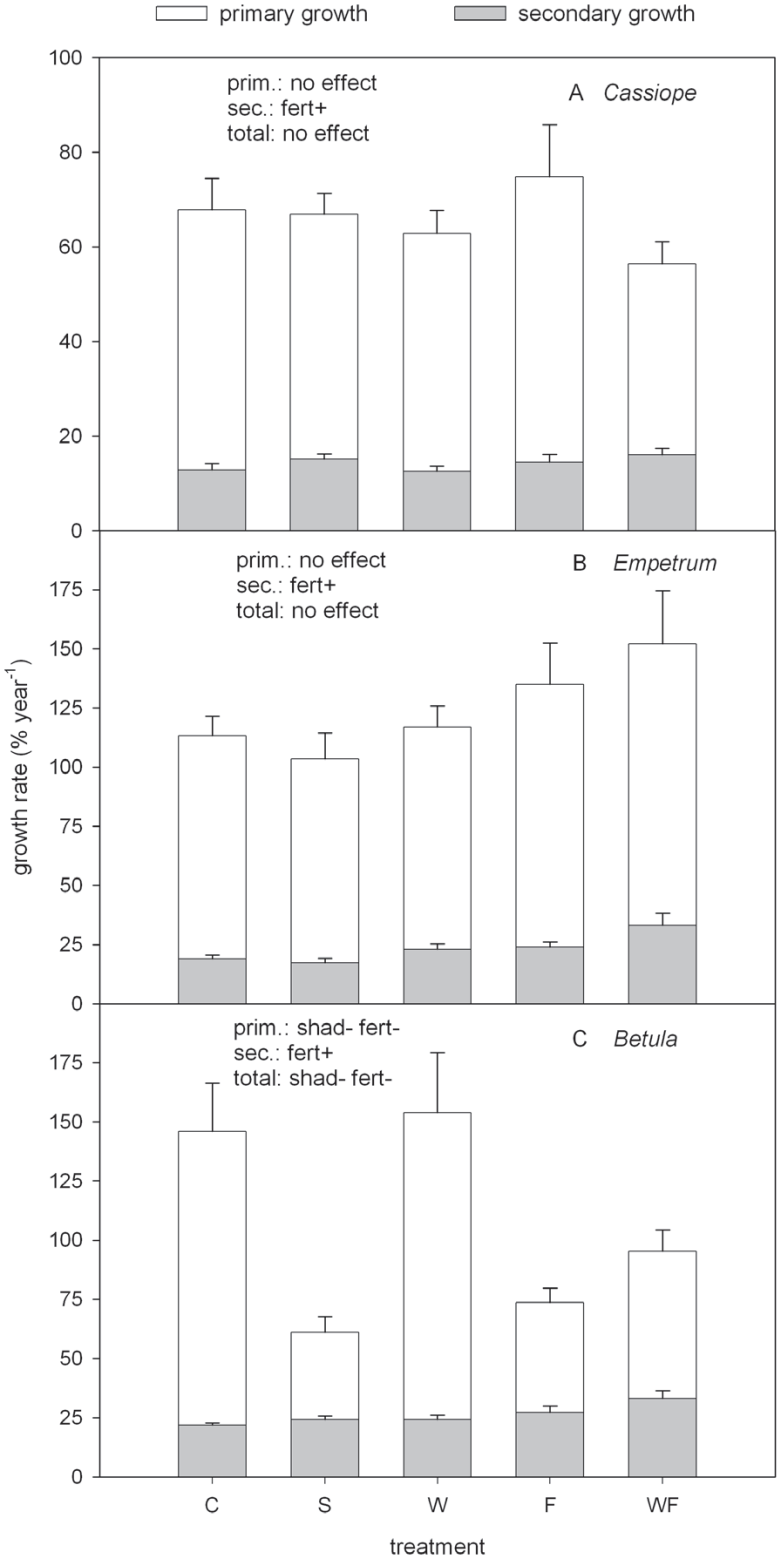
Growth rate of subarctic shrubs in manipulated environment. Growth rate (bars: total aboveground vegetative growth, indicated as ‘total’; white stacks: primary growth i.e. leaves plus apical stem, indicated as ‘prim.’; grey stacks: secondary growth i.e. stem diameter increment, indicated as ‘sec.’; mean+1SE; n = 5–6) of the shrubs *Cassiope tetragona*, *Empetrum hermaphroditum* and *Betula nana* in a subarctic heath in Abisko (Northern Sweden) subjected to 22 years of environmental manipulation: shading (S), warming (W), fertilisation (F), combined warming plus fertilisation (WF). The control is indicated by C. The environmental factors significantly affecting growth are reported on the top left corner of each panel (shad: shading; fert: fertilisation) with the symbols + and − indicating the direction of the response, positive and negative, respectively. Note the different scale between y-axes of panel A and panel B,C.

The long-term environmental manipulations did not affect the total growth of *Cassiope* and *Empetrum* (Fig. 1a,b; Table 1). By contrast, the secondary growth increased under fertilisation for both species (Fig. 1a,b; Table 1). The total growth of *Betula* was significantly reduced (by a factor 2.0–2.4) under shading and fertilisation (Fig. 1c; Table 1). For *Betula*, the primary growth presented significant trends similar as for the total growth, whereas the secondary growth increased under fertilisation (Fig. 1c; Table 1).

**Table 1:** Results of ANOVAs on the growth rates (primary, secondary and total), shrub height, apical (current year's stem) increment and shrub length of the shrubs *Cassiope tetragona*, *Empetrum hermaphroditum* and *Betula nana* at a subarctic heath in Abisko (Northern Sweden) after 22 years of environmental manipulation.

effect	primary growth		secondary growth		total growth		shrub height		apical increment		shrub length	
	*F*	*P*	*F*	*P*	*F*	*P*	*F*	*P*	*F*	*P*	*F*	*P*
*Cassiope*												
shading	0.14^a^	0.71	2.15^a^	0.16	0.01^a^	0.91	<0.01^b^	0.96	4.58^a^	0.045	5.89^a^	0.025
warming	1.96^c^	0.17	0.30^c^	0.58	0.94^c^	0.17	7.05^a^	0.016	6.63^c^	0.014	5.55^c^	0.024
fertilisation	0.46^c^	0.50	4.32^c^	0.044	0.10^c^	0.75	2.77^a^	0.11	0.77^c^	0.39	<0.01^c^	0.95
warm×fert	0.81^c^	0.37	0.54^c^	0.47	0.72^c^	0.40	<0.01^a^	0.99	1.94^c^	0.17	0.35^c^	0.56
*Empetrum*												
shading	0.35^a^	0.56	0.45^a^	0.51	0.57^a^	0.46	2.08^b^	0.15	0.34^a^	0.57	0.19^a^	0.66
warming	0.04^c^	0.84	2.74^c^	0.11	0.52^c^	0.48	17.75^a^	<0.001	2.73^c^	0.11	5.46^c^	0.025
fertilisation	0.93^c^	0.34	4.70^c^	0.036	1.45^c^	0.24	48.23^a^	<0.001	4.38^c^	0.043	4.10^c^	0.050
warm×fert	0.06^c^	0.81	0.04^c^	0.85	0.26^c^	0.61	4.53^a^	0.046	1.27^c^	0.27	1.46^c^	0.23
*Betula*												
shading	6.00^d^	0.014	1.42^d^	0.27	12.73^d^	<0.01	0.30^b^	0.58	<0.01^d^	1.00	0.57^d^	0.48
warming	0.41^e^	0.53	2.84^e^	0.11	0.74^e^	0.40	11.27^a^	<0.01	1.96^e^	0.18	1.15^e^	0.30
fertilisation	18.52^e^	<0.001	9.15^e^	<0.01	14.51^e^	<0.01	5.39^a^	0.031	3.30^e^	0.088	3.56^e^	0.078
warm×fert	0.09^e^	0.76	0.56^e^	0.46	0.16^e^	0.69	0.15^a^	0.70	0.59^e^	0.45	0.59^e^	0.45

a : degrees of freedom of between group variation or effect (df effect) of 1 and degrees of freedom of within group variation or residuals (df residuals) of 20;

b : df effect = 1, df residuals = 10;

c : df effect = 1, df residuals = 40;

d : df effect = 1, df residuals = 8;

e : df effect = 1, df residuals = 16.

Samples from the control treatment showed that the total growth rate was largest for *Betula* (∼130% year^−1^), intermediate for *Empetrum* (∼110% year^−1^) and lowest for *Cassiope* (∼70% year^−1^), and that the primary growth represented 80–85% of the total growth for each species (Fig. 1a,b,c). Leaf production accounted for the large majority of primary growth: 85% for *Cassiope* and *Empetrum* and 98% for *Betula*.

### Shrub height and length

**Figure 2:**
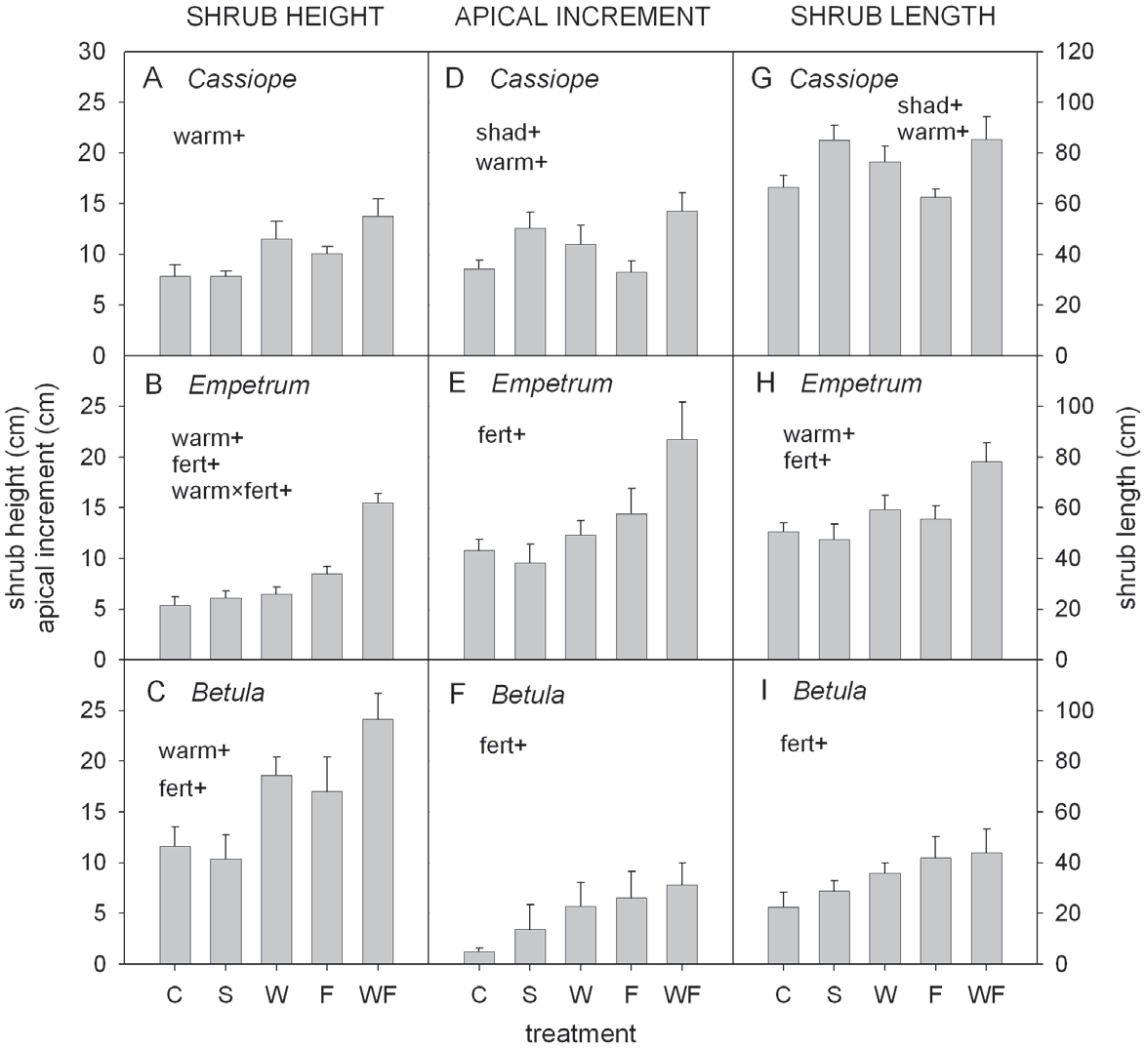
Shrub height, shrub length and apical (current year's stem) increment of subarctic shrubs in manipulated environment. Values (mean+1SE; n = 5–6) of shrub height (left panels), apical increment (central panels) and shrub length (right panel) of three subarctic heath shrubs (*Cassiope tetragona*, *Empetrum hermaphroditum* and *Betula nana*) in Abisko (Northern Sweden) subjected to 22 years of environmental manipulation (shading S, warming W, fertilisation F, combined warming plus fertilisation WF) against the control (C). The environmental factors significantly affecting growth are reported on the upper part of each panel (shad: shading; warm: warming; fert: fertilisation, and warm×fert: warming×fertilisation) with the symbols + and − indicating the direction of the response (positive and negative, respectively).

Shrub height was positively affected by warming for each species and by fertilisation for *Empetrum* and *Betula* (Fig. 2a,b,c; Table 1). Increase in height was of particular relevance in the warming plus fertilisation treatment (WF), where shrubs were 2–3 times taller than in the control (Fig. 2a,b,c). Shading had no effect on shrub height (Fig. 2a,b,c; Table 1). The impact of the environmental manipulations on shrub length was similar to the impact on shrub height (Fig. 2g,h,i). In contrast to shrub height, shrub length of *Cassiope* was positively affected by shading (Fig. 2g; Table 1) which relates to a significant increase in the number and length of the branches (see below).

### Branch number, branch length and apical increment

**Figure 3:**
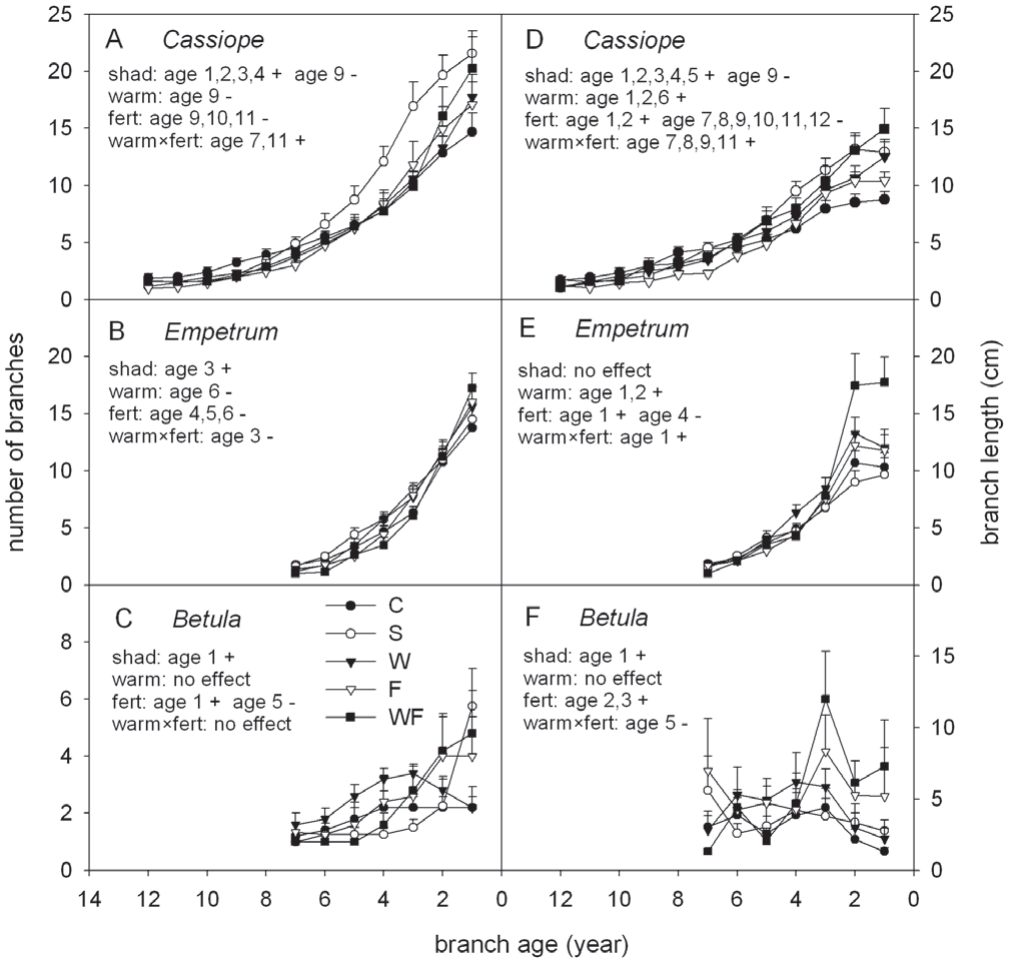
Branch number and length (according to age) of subarctic shrubs in manipulated environment. Number and length of the branches vs. branch age (means+1SE; n = 5–6) of the shrubs *Cassiope tetragona*, *Empetrum hermaphroditum* and *Betula nana* at a subarctic heath in Abisko (Northern Sweden) after 22 years of environmental manipulation. Text on the top left indicates the environmental factors with a significant impact (shad: shading; warm: warming; fert: fertilisation, and warm×fert: warming×fertilisation), their direction (+: positive, −: negative) and the age of the branches affected. Note the different scale between y-axes of panel A,B,D,E and panel C,F.

Shading exerted mainly a positive impact on the number and length of the youngest branches, particularly for *Cassiope* (Fig. 3a,d). Fertilisation had mainly a negative impact on the number and length of old branches of *Empetrum* and, particularly, *Cassiope* and a positive impact on the length of the youngest branches (up to 3 year-old) of all species (Fig. 3). The impact of warming was minor and affecting only *Cassiope* and *Empetrum*, with reduced branch numbers in few old cohorts and increased branch length in few young cohorts (Fig. 3a,b,d,e). The *F* and *p* values of the ANOVA analyses are reported in Table S1.

The apical increment of *Cassiope* was positively affected by shading and warming, whereas the apical increment of *Empetrum* and *Betula* was positively affected by fertilisation (Fig. 2d,e,f; Table 1). Overall, the number of branches was similar for *Cassiope* and *Empetrum* but much lower (a factor of 2–4) for *Betula* (Fig. 3a,b,c). *Cassiope* and *Empetrum* presented branch length larger than *Betula* for young cohorts but similar branch length for older cohorts (Fig. 3d,e,f).

### Species coverage

**Figure 4:**
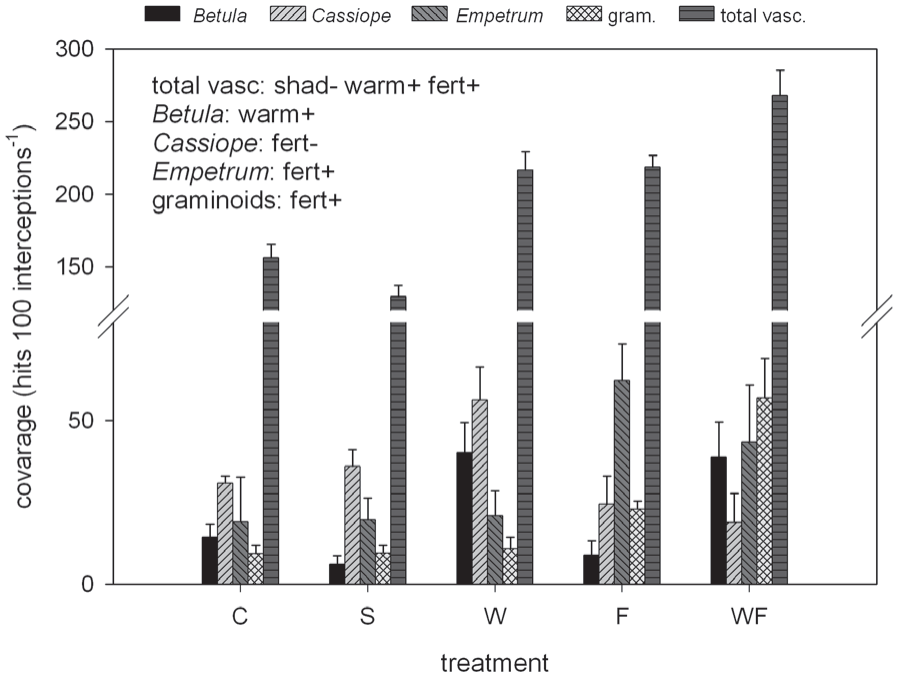
Species coverage in subarctic heath under environmental perturbations. Coverage (mean+1SE; n = 6) of the shrubs *Cassiope tetragona*, *Empetrum hermaphroditum* and *Betula nana*, the graminoids (gram.) and of the total vascular species (total vasc.) at a subarctic heath in Abisko (Northern Sweden) after 22 years of environmental manipulation: shading (S), warming (W), fertilisation (F), combined warming plus fertilisation (WF). Text on the top left indicates the environmental factors with a significant impact on coverage (shad: shading; warm: warming; fert: fertilisation, and warm×fert: warming×fertilisation) and their direction (+: positive, −: negative).

Fertilisation had a positive impact on the coverage of graminoids and *Empetrum* and negative on the coverage of *Cassiope*, whereas warming had a positive effect on *Betula* (Fig. 4; Table 2). Overall, the total vascular cover was positively affected by warming and fertilisation and negatively affected by shading (Fig. 4; Table 2). Graminoids increased by a factor of 6 in the WF treatment (Fig. 4).

**Table 2:** Results of ANOVAs on the coverage of the dwarf-shrubs *Cassiope tetragona* and *Empetrum hermaphroditum*, the low shrub *Betula nana*, the graminoids and the total vascular species at a subarctic heath in Abisko (Northern Sweden) after 22 years of environmental manipulation.

plant type	shading^a^		warming^b^		fertilisation^b^		warm×fert^b^	
	*F*	*P*	*F*	*P*	*F*	*P*	*F*	*P*
*Cassiope*	0.71	0.42	0.02	0.89	8.88	<0.01	1.35	0.26
*Empetrum*	0.93	0.34	0.35	0.56	5.35	0.03	0.52	0.48
*Betula*	2.51	0.14	13.02	<0.01	1.00	0.33	1.00	0.33
graminoids	<0.01	0.97	2.39	0.14	25.36	<0.001	1.99	0.17
total vascular	3.92	0.076	15.95	<0.001	17.14	<0.001	0.19	0.67

a : degrees of freedom of between group variation or effect (df effect) of 1 and degrees of freedom of within group variation or residuals (df residuals) of 10;

b : df effect = 1 and df residuals = 20.

## Discussion

Our expectations were only partially confirmed, with differences among treatments and species. Both hypotheses were overall valid under fertilisation but not under warming. For shading, the expectations were confirmed for *Empetrum* and *Betula* only partially and were not confirmed for *Cassiope*. Besides the growth patterns, the study showed that the investigated shrubs developed a significant plasticity over the longer term.

### Hypothesis 1: growth responses change over the longer term

#### Fertilisation

As expected, the effect of fertilisation became less favourable over the longer term for each species. The effect of fertilisation on *Empetrum* and *Cassiope* changed from positive to non-significant (Table 3). This reveals that the positive effect of nutrient addition on the growth of evergreen dwarf-shrubs was transient at our experiment because of the concomitant positive effect of fertilisation on graminoids. A progressive shrub decline concurrent to an increase in graminoids has previously been observed in other subarctic and low arctic fertilised heaths [28], [29]. Furthermore, the growth of evergreen dwarf-shrubs degenerated in tussock tundra after 3 and 9 years of fertilisation because of the progressive increase in competition with *Betula* [9]. However, our study is the first to show that after more than two decades of treatment, aboveground growth of evergreen dwarf-shrubs is not suppressed and ancillary positive growth impacts are still recorded (e.g. increase in *Empetrum* coverage, Fig. 4). This is likely due to the shade tolerance of *Cassiope* and *Empetrum* and to morphological plasticity (see below). *Betula* showed a different dynamics as the effect of fertilisation changed from non-significant to strongly negative (i.e. halving of the growth rate). Despite an important plastic response (see below), *Betula* was suppressed over the longer term because the competition with graminoids was particularly severe for this species characterized by low shade tolerance. However, *Betula* might suffer competition (or other growth limitations) even in control conditions at our site and fertilisation likely exacerbates a natural constrained growth. This is supported by comparing the growth of *Betula* at the experimental site with the growth of *Betula* at other heath sites [27] and by the fact that the growth of fertilised *Betula* was stimulated after 8 years of treatment in a more open tundra heath despite an even larger increase in graminoids abundance [28].

**Table 3:** Synthesis of the significant impact of shading (S), warming (W), fertilisation (F) and their interactions on the growth of the tundra shrubs *Cassiope tetragona*, *Empetrum hermaphroditum* and *Betula nana*.

year^a^	aboveground growth response		shrubs species^b^			reference^c^
	variable	meristem (organ)	*Cassiope*	*Empetrum*	*Betula*	
3	mass per shoot	primary (leaf)	F+	n.a.	n.a.	[19]
5	biomass ratio^d^	primary (leaf, stem)	F+	n.a.	n.a.	[21]
5	biomass unit ground	primary plus secondary (leaf, stem)	W+, F+	W×F+	n.a.	[5], [21]
6	mass per shoot	primary (leaf)	n.a.	S-	S-	[20]
6	shoot density	primary (stem)	n.a.	F+, W×F+	no effect	[20]
10	biomass unit ground	primary (leaf)	S-	F+	S−	[5]
10	biomass unit ground	primary plus secondary (stem)	no effect	no effect	S−	[5]
10	biomass unit ground	primary plus secondary (leaf, stem)	no effect	F+	S−	[5]
22	growth rate	primary (leaf, stem)	no effect	no effect	S−, F−	this study
22	growth rate	secondary (stem)	F+	F+	F+	this study
22	growth rate	primary plus secondary (leaf, stem)	no effect	no effect	S−, F−	this study

+: positive impact; −: negative impact; no effect: manipulations had no significant impact on the growth variable; W×F− and W×F+: lower and higher effects in the combined treatment, respectively, than expected from the single treatments alone; n.a.: no data available;

a : years of treatment at the time of measurement;

b : non-significant effects are not listed;

c : data refer only to the experimental site investigated in this study (the tree-line heath of Paddustieva, Abisko, Northern Sweden) and are derived from earlier published papers and the current work, and

d : green biomass as ratio of grey stem biomass.

#### Shading

Despite some acclimation to shade (e.g. increased leaf nitrogen and chlorophyll [21]), the growth of *Cassiope* and *Empetrum* showed some negative responses in the first 10 years of shading treatment (Table 3). Over the longer term, contrary to our expectations, negative effects disappeared as the response of the growth rate of *Cassiope* and *Empetrum* to shade was non-significant. *Cassiope* was expected to have a limited shade tolerance as it does not grow in shaded habitats [19]. However, *Cassiope* likely compensated the negative effect of shading by substantial changes in allocation pattern. While maintaining the same growth rate (and thus biomass production), shaded *Cassiope* ramets increased greatly in branch numbers and branch length (Fig. 3a,d), hence increasing photosynthesising surface and light interception. *Empetrum* is likely to be shade tolerant (it grows in the understory of taller tundra shrubs and boreal forests [21]) and only very minor changes in morphology were observed over the longer term. On the other hand, the response of *Betula* to shading confirmed our expectations for this species. The reduction of aboveground growth of shaded *Betula* was one of the most significant responses recorded in our study and it was related to a strong reduction in leaf production. This confirms that the negative response of *Betula* leaves observed earlier at the same plots was not transient (Table 3). On the other hand, the observed continuous growth decline was not associated with alteration in *Betula* cover (Fig. 4). Despite the well known low tolerance of *Betula* to shade [30], [31], some compensatory processes might have played a role over the longer term and avoided complete suppression. For instance, shaded *Betula* might have partially benefitted from a reduction in total vascular cover (Fig. 4) or from a reduction in the stem turnover, which is stimulated in shaded *Betula* [9] and is perhaps responsible for the transient reduction in stem biomass recorded earlier in our plots (Table 3).

#### Warming

Contrary to our expectations, the effect of warming on aboveground growth was non-significant after 22 years of treatment. Despite the consensus on the positive direct effect of warming on the growth of arctic plants, our findings suggest that physiological processes limiting net biomass production (e.g. respiration, stem turnover; [9], [11]) do not acclimate over the longer term. Alternatively, other factors might limit growth under long-term warming [11]. For instance, nutrient limitation might be important in our warmed plots due to increased vascular cover and competition (Fig. 4). On the other hand, heat stress might occur on warmed plants during warm summer days [11], as observed for *Ledum palustre* in the Low Arctic [9] and in *Salix arctica* in the Mid Arctic [32]. *Empetrum* is likely to be particularly sensitive to heat stress as it was favoured by warming level of 2.5°C and not by warming level of 4°C at our site after 6 years of treatment [20]. Our results indicate thus that the positive effect of warming on *Cassiope* recorded after 5 years of treatment was transient (Table 3). Transient positive responses to warming have been observed in other short-term tundra warming experiments and associated with temporary increases in mineralization or use of stored resources [11].

### Hypothesis 2: responses of primary and secondary growth differ according to morphology

#### Fertilisation

Fertilised ramets of the three species showed a similar pattern with non-significant variation or decrease in primary growth and increase in secondary growth (Fig. 1). For *Empetrum* and *Betula*, this pattern was accompanied by a significant increase in shrub height, total shrub length and length of the youngest branches. For these two species our expectations were therefore confirmed. In presence of a lush graminoid canopy, *Empetrum* and *Betula* (procumbent at the site) grew more vertically and explored more lateral space. A more erected and large posture requires more resources to reinforce the stem mechanical strength, implying enhanced secondary growth. This typical morphological plastic reaction prevented these shrubs from being completely confined in the shaded understory. For *Empetrum*, such response likely avoided reduction in the aboveground growth. For *Betula*, such plastic reaction was likely not enough to maintain the same C assimilation as in the control, resulting in fewer resources available for primary growth and in an overall growth reduction. *Cassiope* is likely to have a similar pattern as for *Empetrum* but less marked. In fact, for *Cassiope*, the significant increase in secondary growth was coupled to a non-significant increase in shrub height (p = 0.11) (Table 1).

#### Shading

Our expectations were confirmed for *Empetrum*, whose growth pattern and morphology were both unaffected by shading. For *Betula*, we did not expect uncoupling in the response of primary and secondary growth as the morphology of *Betula* did not change in shaded plots. However, the prolonged light attenuation significantly decreased *Betula* leaf production, impairing the relationship between primary and secondary growth. *Cassiope* showed a less clear pattern with no increase in secondary growth and substantial increase in total length and branching. However, the fact that the shaded *Cassiope* ramets had low stature (Fig. 2b) probably resulted in procumbent *Cassiope* branches laid on the moss mat, thus requiring less mechanical support from the stem.

#### Warming

Warmed ramets showed unaffected primary and secondary growth but increased shrub height and (for *Cassiope* and *Empetrum*) increased shrub length and branch length of the youngest branches. This was unexpected. It is possible that the morphological changes under warming were the result of an overall improved (micro)environment rather than the result of the competition for light as in the fertilised plots. Such supposition is coherent with the species coverage results, which showed no negative impact of warming, increase in total vascular cover and no impact on graminoids (Fig. 4).

#### Overall

Previous short term (<10 years) studies on tundra concluded that evergreen dwarf-shrubs have low developmental plasticity, conservative secondary growth and tend to become subcanopy species [9], [26], [33]. Our study reveals that evergreen dwarf-shrubs can show opposite dynamics over the longer term and posses an overall important plasticity. As acclimation normally occurs through formation of new tissue, it is indeed plausible that slow growing species need long time to acclimate [9], [34]. Furthermore, our study showed that apical increment presented a different response than primary growth for each treatment and species. This because length increment does not necessarily correlate to biomass increment in arctic shrubs [25], [27]. Such uncoupling calls for caution when inferring growth responses of tundra shrub to environmental manipulations from apical increment only.

## Materials and Methods

### Experimental set up

#### Study site

The study took place at a tree-line heath on sloping terrain (20–30%) at 450 m a.s.l. at Abisko (68°21′N, 18°49′E), in Northern Sweden. The region has a subarctic montane climate, with mean annual temperature and precipitation of −1.0°C and 304 mm, respectively, and the growing season lasting from early-mid June until late August-early September [25], [35]. The site is an evergreen dwarf-shrub community dominated by *Cassiope tetragona* and by the co-dominant *Empetrum hermaphroditum*. *Betula nana* is one of the most common deciduous shrubs. Graminoids and forbs are also present, as well as nonvascular plants which form a continuous mat [20]. Bedrock consists of base-rich mica schists [19]. The soil has pH of 7.1 (typical for ecosystems with similar bedrock and topography in the region [36]), an organic layer of about 15 cm and is well drained [25], [37], [38]. No specific permits were required for the described field studies, as the location is not privately-owned and not protected and the field studies did not involve endangered or protected species.

#### Environmental manipulations

The experiment started in 1989 in an area of about 400 m^2^. It consisted of eight treatments replicated in six blocks: control, low warming, high warming, shading, fertilisation, fertilisation plus low warming, fertilisation plus high warming and fertilisation plus shading [13]. In this study, we investigated five key treatments: shading (S), high warming (W), fertilisation (F), fertilisation plus high warming (WF) and control (C). Temperature was enhanced by small (1.2×1.2 m, 50 cm high) dome-shaped open top greenhouses of polyethylene film (0.05 mm) supported by PVC tubes. Greenhouses were in place every year from early June (just after snowmelt) until end of August-early September (leaf fall) enhancing the summer air and soil temperature by 3.9°C and 1.2°–1.8°C, respectively. The greenhouses did not provoke critical side effects on plant growth because: (1) they caused only minor and non-significant reduction in relative soil water content (<6%) as the sloping terrain permitted lateral water movement [19], [21]; (2) they did not change significantly the air humidity (<3%); (3) they only led to a 9% reduction in photosynthetically active radiation [19], [21]; (4) they did not affect the snow cover as they were not in place in winter and (5) their sheltering effect had a minor impact on shrub morphology as in tundra heath the effect of wind exposure on the shrub structure is much more relevant in winter [7], [39]. Furthermore, the greenhouses (resting on the shrub canopy and opened at the top) do not impact the presence of small herbivores (e.g. insects, rodents). Reindeer grazing and moose browsing is generally limited to periods out of the growing season (e.g. reindeers normally do not stay at the tree-line during summer) and are not affected by our manipulative experiment, which is in place only between June and August. The shading was obtained with hessian (jute) fabric, arranged in the same way and in the same period as the polyethylene film of the greenhouses. Hessian fabric reduced the light by 64% without significant effect on air humidity and temperature [19], [21]. Fertilisation (10.0 g m^−2^ N, 2.6 g m^−2^ P and 9.0 g m^−2^ K, in the form of NH_4_NO_3_, KH_2_PO_4_ and KCl) occurred once per year after snow melt in June (except in 1998, half amount, and in 1993 and 2001, not applied). The dose of N applied was considered similar in magnitude to potential N release from the soil of tundra ecosystems under global change scenario [15].

#### Sampling and processing of shrub ramets

Sampling took place in mid-late August 2010, after 22 years of experimental manipulation. Individual ramets (i.e. aboveground stem with all lateral branches) of *Cassiope* and *Empetrum* (two ramets) and *Betula* (one ramet) were collected for each block and treatment (n = 6). However, for *Betula*, only five replicates were considered in the analysis as most of the ramets of one block were too young or too damaged (i.e. broken stem or branches) to be analyzed. The ramets were sampled in the central part of the experimental plots. This assured that biases due to sampling of ramets supporting biomass outside of the plot or ramets grown into the plots after the treatment began were minor because (i) the colonization rate of these slow growth ericaceous plants is inherently low [19], (ii) the shrub frequency in the area surrounding the plots has been reduced by trampling [40] and (iii) woody functional type as evergreen and multiple-flush deciduous have a high degree of ‘shoot autonomy’ [41], meaning that relocation of assimilates among ramets is limited.

Stem and branches were divided into segment cohorts of the same age. This was done by counting the apical bud scars for *Empetrum* [24], the stem sections with smaller leaves for *Cassiope* [42] and the annual growth rings in thin stem/branch cross-sections after staining with 0.5% phloroglucinol in 10% HCl for *Betula* [26]. Each stem/branch segment cohort was dried at 70°C for 48 hours and leaves were detached. The number of stem/branch segments of each cohort, their aggregated length and dry weight were recorded [24] as well as the weight of the current-year leaves. Ramets of *Empetrum* and *Betula* were 5–7 year-old. Ramets of *Cassiope* were 10–12 year-old.

### Measurement of growth variables

Growth of shrubs was assessed with comparable but refined methods to the ones used to characterize growth responses in the first decade of manipulation, as the latter were too invasive to be repeated (e.g. standing biomass harvest) or had limitations (e.g. lack of assessment of secondary growth and branching pattern). In the current study, shrub growth was determined as primary growth (leaf and apical stem production) and secondary growth, both expressed as percentage of old stem biomass [24], [26], [27], [43], [44]. Data on growth rate were complemented by several measurements of shrub morphology (e.g. shrub height, number and length of branches, total length of stem and branches) and of species coverage. Growth rates were used to evaluate the long-term impact of the environmental manipulations on shrub growth because they provide a direct estimation of plant biomass production. Morphological characteristics were compared to the growth rate estimates to better understand the response pattern of the primary- and secondary growth. The species coverage was used to indirectly infer species abundance. A summary of the growth variables measured, the plant organ and organ age considered and the way the results are expressed (e.g. relative or absolute values, values per plot or per ramet) is reported in Table 4.

**Table 4:** Summary of the shrub growth variables measured in this study, the plant organ and organ age they refer to and the way the results are expressed.

Growth variables	species	organ^a^	age organ (year)	result type
Primary growth	*Cassiope*	leaves plus stem/branches	0^b^	relative to old stem biomass
	*Empetrum*, *Betula*	leaves plus stem/branches	0	relative to old stem biomass
Secondary growth	*Cassiope*	stem/branches	1 to 12	relative to old stem biomass
	*Empetrum*, *Betula*	stem/branches	1 to 7	relative to old stem biomass
Total growth	*Cassiope*	leaves plus stem/branches	0 to 12	relative to old stem biomass
	*Empetrum*, *Betula*	leaves plus stem/branches	0 to 7	relative to old stem biomass
Shrub height	*Cassiope*	stem/branches	no distinction	absolute per plot
	*Empetrum*, *Betula*	stem/branches	no distinction	absolute per plot
Shrub length	*Cassiope*	stem/branches	1 to 12	absolute for each ramet
	*Empetrum*, *Betula*	stem/branches	1 to 7	absolute for each ramet
Branch numbers	*Cassiope*	stem/branches	1 to 12	absolute for each cohort
	*Empetrum*, *Betula*	stem/branches	1 to 7	absolute for each cohort
Branch length	*Cassiope*	stem/branches	1 to12	absolute for each cohort
	*Empetrum*, *Betula*	stem/branches	1 to 7	absolute for each cohort
Apical increment	*Cassiope*	stem/branches	0	absolute for each ramet
	*Empetrum*, *Betula*	stem/branches	0	absolute for each ramet
Plant coverage	*Cassiope*	leaves plus stem/branches	no distinction	absolute per plot
	*Empetrum*, *Betula*	leaves plus stem/branches	no distinction	absolute per plot

a : no distinction was made for stem and branches as they are difficult to differentiate for the clonal species investigated;

b : 0 indicates ‘current year’.

#### Growth rates

We assessed the annual rate of the aboveground vegetative growth as primary-, secondary- and total growth. We determined primary growth rate (% year^−1^) for each ramet as the production of current year's apical biomass (leaves and stem) as a percentage of old standing stem biomass [27]. The data needed to calculate the primary growth were directly available from the harvested material (see above). The stem secondary growth was expressed in the same way but the current-year secondary growth was not directly available from the harvested material. Instead, we derived that following the model of Bret-Harte et al. [26] (see key equations below) who assumed that (i) a stem/branch segment is cylindrical, (ii) the annual increment in stem/branch radius does not vary with the age of the stem/branch segments in the aboveground portion of the ramet, and (iii) the annual increment in stem/branch radius changes under manipulated environmental conditions. For the study species, the performance of the model of Bret-Harte et al. [26] was very good, with average slopes of the regression modelled vs. measured standing stem biomass *M* of 0.90–1.1 and *r* ^2^>0.94 [45]. The total growth rate was calculated as the sum of the primary and secondary growth rate.

The calculation of the secondary growth of individual ramets at yearly basis is summarized in four steps (for details see [26]). (i) The mass (*m*) of a stem/branch segment cohort of a given age *n* (in years) of an individual ramet is estimated as:
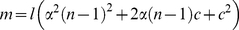
(1)where *l* is the length of the stem/branch segment cohort, *c* equals (*m*/*l*)^1/2^ of the current-year stem and α the slope of a linear relationship (*m*/*l*)^1/2^ vs. *n*. (ii) The annual mass increment due to secondary growth (*Δm*) of a stem/branch segment cohort equals:
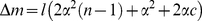
(2)(iii) The mass (*M*) of an individual ramet is calculated as the sum of *m* for all the segment cohort age classes and the annual mass increment due to secondary growth (Δ*M*) as the sum of *Δm* for all the segment cohort age classes. (iv) The annual stem secondary growth rate equals Δ*M*/*M*.

#### Shrub height and length

The height of nine randomly selected shoots of *Cassiope*, *Empetrum* and *Betula* was measured in two 25×50 cm rectangulars within the central area of each plot in mid-late August 2010 and averaged for each plot (n = 6). The height was measured with a ruler as the perpendicular height from shoot apex to ground. Shrub length refers to the total length of stem and branches of each ramet of *Cassiope*, *Empetrum* and *Betula* sampled for the determination of the growth rate (see above).

#### Branch number, branch length and apical increment

Branch number and branch length of each ramet of *Cassiope*, *Empetrum* and *Betula* were derived separately for each age class composing the ramet (in years) from the ramets sampled for the determination of the growth rate (see above). Branch length refers to the total aggregated length for a given branch age class. The length of the current year's branches is defined as apical increment. As young stem and young branches are difficult to differentiate for the clonal species investigated, all woody segments of a given age class were considered as branches in these assessments.

#### Species coverage

Species coverage was measured in mid-late August 2010 with the pin-point method in the same two rectangulars (25×50 cm) per plot investigated for shrub height. A pin was passed vertically at 100 points (5 cm spaced) and all matter touched by the pin was recorded as a hit [46]. Vascular vegetation was recorded at species level, nonvascular vegetation was lumped in bryophytes and lichens, whereas attached or unattached dead tissue was recorded as litter. In this study, we present coverage data for the model shrub species *Cassiope*, *Empetrum* and *Betula*, for the lumped graminoids group and for the total vascular plant cover.

### Statistics

The impact of manipulated environmental factors (warming, fertilisation, shading) was assessed with analysis of variance. Due to the incomplete factorial design, the analysis was conducted separately for (i) shading and (ii) warming and fertilisation [13]. The response to shading was tested with a one-way ANOVA, whereas the response to warming and fertilisation with a two-way ANOVA with interaction between warming and fertilisation. The block factor was not considered because preliminary analyses showed that it had no effect on the dependent variables. If prerequisites for analysis of variances (normality, checked with Shapiro test, and homoscedasticity, checked with Bartlett test) were not met, we performed a Kruskal-Wallis test instead of one-way ANOVA or repeated the same analysis after transformation (log*x* and in few cases *x* ^1/2^ or *x* ^−1^) for two-way ANOVA, as no standard non-parametric test fitted our design and transformed data fulfilled the ANOVA prerequisites. Transformation was needed for: (i) growth rates of *Cassiope* and *Empetrum*, (ii) total height of each species, (iii) total length and apical increment of *Cassiope* and *Empetrum*, (iv) coverage of *Cassiope*, *Betula* and graminoids, and (v) datasets on branch number and branch length (see details in Table S1).

Dependent variables tested with the ANOVA analysis were growth rates (primary, secondary and total), shrub height, shrub length, species coverage, apical increment, branch number and length. Primary and secondary growth were tested separately as they proved to be uncorrelated (tested with Pearson's correlation) for any treatment and species. If more within-plot measures of the dependent variable were available per plot (e.g. for growth rates of *Cassiope* and *Empetrum*), a nested level was added to the ANOVAs. Treatment effects on branch number and length were tested separately for each species and age class, except for 7 year-old branches of *Empetrum* and *Betula* which were not analyzed because of the few replicates available. All analyses were performed in R version 2.12.2 (R Development Core Team 2011).

## Supporting Information

Table S1 Results of ANOVAs (F and P values) on the number and length of branches of cohort of the same age (up to 12 year-old) of the shrubs *Cassiope tetragona*, *Empetrum hermaphroditum* and *Betula nana* at a subarctic heath in Abisko (Northern Sweden) after 22 years of environmental manipulation.
(DOC)
