# Supplementary material for: Twenty-Two Years of Warming, Fertilisation and Shading of Subarctic Heath Shrubs Promote Secondary Growth and Plasticity but Not Primary Growth
Source: PLoS One. 2012 Apr 12;7(4):e34842. doi: 10.1371/journal.pone.0034842 (PMC3325270; doi:10.1371/journal.pone.0034842)
Supplement: Table S1 — Results of ANOVAs (F and P values) on the number and length of branches of cohort of the same age (up to 12 year-old) of the shrubs Cassiope tetragona, Empetrum hermaphroditum and Betula nana at a subarctic heath in Abisko (Northern Sweden) after 22 years of environmental manipulation. (DOC) [file pone.0034842.s001.doc]

Table S1. Results of ANOVAs on the number and length of branches of branch cohort of the same age (up to 12 year-old) of the shrubs *Cassiope tetragona*, *Empetrum* *hermaphroditum* and *Betula* *nana* at a subarctic heath in Abisko (Northern Sweden) after 22 years of environmental manipulation.

| effect | *Cassiope* | | | | *Empetrum* | | | | *Betula* | | | |
| --- | --- | --- | --- | --- | --- | --- | --- | --- | --- | --- | --- | --- |
|  | branch number | | branch length | | branch number | | branch length | | branch number | | branch length | |
|  | F | P | F | P | F | P | F | P | F | P | F | P |
| *1 year-old* | | | | | | | | | | | | |
| shading | 9.87 | <0.01 | 8.79 | <0.01 | 0.18 | 0.67 | 0.96 | 0.34 | 8.32 | 0.023 | 4.27 | 0.078 |
| warming | 2.28 | 0.14 | 14.57^T^ | <0.001^T^ | 1.27 | 0.27 | 3.20^T^ | 0.081^T^ | 0.13 | 0.72 | 0.21^T^ | 0.65^T^ |
| fertilization | 1.37 | 0.25 | 3.91^T^ | 0.055^T^ | 2.04 | 0.16 | 4.42^T^ | 0.042^T^ | 4.02 | 0.062 | 2.99^T^ | 0.10^T^ |
| warm × fert | <0.01 | 0.99 | 0.03^T^ | 0.867^T^ | 0.05 | 0.83 | 1.58^T^ | 0.22^T^ | 0.13 | 0.72 | 0.12^T^ | 0.74^T^ |
| *2 years-old* | | | | | | | | | | | | |
| shading | 8.42 | <0.01 | 10.34 | <0.01 | 0.04 | 0.85 | 1.29 | 0.27 | <0.01 | 0.96 | 0.96 | 0.39 |
| warming | 0.22 | 0.64 | 5.01^T^ | 0.031^T^ | 0.12 | 0.73 | 3.13^T^ | 0.085^T^ | 0.16 | 0.69 | 0.60 | 0.45 |
| fertilization | 1.67 | 0.20 | 4.49^T^ | 0.040^T^ | 0.03 | 0.86 | 2.40^T^ | 0.13^T^ | 2.61 | 0.13 | 7.96 | 0.012 |
| warm × fert | 0.03 | 0.86 | 0.03^T^ | 0.87^T^ | 0.49 | 0.49 | 0.11^T^ | 0.74^T^ | 0.04 | 0.84 | <0.01 | 0.98 |
| *3 years-old* | | | | | | | | | | | | |
| shading | 7.87 | 0.011 | 7.23 | 0.014 | 7.18 | 0.014 | <0.01 | 0.96 | 2.01 | 0.20 | 0.10 | 0.76 |
| warming | 0.42^T^ | 0.52^T^ | 2.25 | 0.14 | 0.09 | 0.77 | 0.08^T^ | 0.77^T^ | 2.19^T^ | 0.16^T^ | 1.25 | 0.28 |
| fertilization | 0.01^T^ | 0.90^T^ | 1.23 | 0.27 | <0.01 | 0.95 | 0.02^T^ | 0.88^T^ | 0.54^T^ | 0.47^T^ | 4.86 | 0.043 |
| warm × fert | 0.22^T^ | 0.64^T^ | 0.09 | 0.76 | 4.67 | 0.037 | 1.28^T^ | 0.26^T^ | 0.29^T^ | 0.60^T^ | 0.24 | 0.63 |
| *4 years-old* | | | | | | | | | | | | |
| shading | 7.59 | 0.012 | 8.60 | <0.01 | 1.77 | 0.20 | 0.02 | 0.89 | 1.86 | 0.21 | 0.96 | 0.39 |
| warming | 0.11^T^ | 0.74^T^ | 2.54 | 0.12 | 0.02 | 0.89 | 0.65^T^ | 0.42^T^ | 0.19^T^ | 0.67^T^ | 0.27^T^ | 0.61^T^ |
| fertilization | 0.04^T^ | 0.84^T^ | 0.47 | 0.49 | 4.29 | 0.045 | 5.22^T^ | 0.028^T^ | 2.35^T^ | 0.15^T^ | 0.04^T^ | 0.85^T^ |
| warm × fert | 0.24^T^ | 0.63^T^ | 0.03 | 0.87 | 2.75 | 0.11 | 0.85^T^ | 0.36^T^ | 2.35^T^ | 0.15^T^ | 1.02^T^ | 0.33^T^ |
| *5 years-old* | | | | | | | | | | | | |
| shading | 2.59 | 0.12 | 3.12 | 0.093 | 1.02 | 0.32 | 0.42 | 0.53 | 0.34 | 0.66 | 0.14 | 0.80 |
| warming | 0.27^T^ | 0.61^T^ | 2.61^T^ | 0.11^T^ | 0.24 | 0.63 | 0.85 | 0.36 | 0.17^T^ | 0.68^T^ | 0.02 | 0.88 |
| fertilization | <0.01^T^ | 0.98^T^ | <0.01^T^ | 0.99^T^ | 3.21 | 0.081 | 1.34 | 0.25 | 5.12^T^ | 0.038^T^ | 0.08 | 0.77 |
| warm × fert | <0.01^T^ | 0.97^T^ | 0.26^T^ | 0.61^T^ | <0.01 | 0.99 | 0.25 | 0.62 | 2.71^T^ | 0.12^T^ | 4.09 | 0.060 |
| *6 years-old* | | | | | | | | | | | | |
| shading | 0.38 | 0.55 | 1.06 | 0.32 | 0.09 | 0.76 | 0.39 | 0.54 | 0.20 | 0.77 | 0.67 | 0.44 |
| warming | 0.03 | 0.87 | 2.78^T^ | 0.10^T^ | 4.42 | 0.044 | 0.13 | 0.73 | 0.32 | 0.58 | 0.34 | 0.57 |
| fertilization | 0.21 | 0.65 | 1.09^T^ | 0.30^T^ | 4.36 | 0.046 | 0.24 | 0.63 | 2.25 | 0.16 | 0.03 | 0.86 |
| warm × fert | 0.87 | 0.36 | 0.09^T^ | 0.77^T^ | <0.01 | 0.97 | 0.02 | 0.90 | 1.18 | 0.30 | 0.19 | 0.67 |
| *7 years-old* | | | | | | | | | | | | |
| shading | 0.20 | 0.66 | <0.01 | 0.97 | n.a. | n.a. | n.a. | n.a. | n.a. | n.a. | n.a. | n.a. |
| warming | <0.01 | 0.99 | 0.12 | 0.73 | n.a. | n.a. | n.a. | n.a. | n.a. | n.a. | n.a. | n.a. |
| fertilization | 2.04 | 0.16 | 4.33 | 0.044 | n.a. | n.a. | n.a. | n.a. | n.a. | n.a. | n.a. | n.a. |
| warm × fert | 3.30 | 0.077 | 6.19 | 0.017 | n.a. | n.a. | n.a. | n.a. | n.a. | n.a. | n.a. | n.a. |
| *8 years-old* | | | | | | | | | | | | |
| shading | 0.62 | 0.44 | 2.87 | 0.11 | n.a. | n.a. | n.a. | n.a. | n.a. | n.a. | n.a. | n.a. |
| warming | 0.64^T^ | 0.43^T^ | 0.31 | 0.58 | n.a. | n.a. | n.a. | n.a. | n.a. | n.a. | n.a. | n.a. |
| fertilization | 2.18^T^ | 0.15^T^ | 3.71 | 0.062 | n.a. | n.a. | n.a. | n.a. | n.a. | n.a. | n.a. | n.a. |
| warm × fert | 2.56^T^ | 0.12^T^ | 7.95 | <0.01 | n.a. | n.a. | n.a. | n.a. | n.a. | n.a. | n.a. | n.a. |
| *9 years-old* | | | | | | | | | | | | |
| shading | 3.89 | 0.063 | 4.94 | 0.038 | n.a. | n.a. | n.a. | n.a. | n.a. | n.a. | n.a. | n.a. |
| warming | 3.18^T^ | 0.082^T^ | 1.16^T^ | 0.29^T^ | n.a. | n.a. | n.a. | n.a. | n.a. | n.a. | n.a. | n.a. |
| fertilization | 8.15 ^T^ | <0.01^T^ | 3.37^T^ | 0.074^T^ | n.a. | n.a. | n.a. | n.a. | n.a. | n.a. | n.a. | n.a. |
| warm × fert | 0.66^T^ | 0.42^T^ | 5.93^T^ | 0.020^T^ | n.a. | n.a. | n.a. | n.a. | n.a. | n.a. | n.a. | n.a. |
| *10 years-old* | | | | | | | | | | | | |
| shading | 2.61 | 0.12 | 0.52 | 0.48 | n.a. | n.a. | n.a. | n.a. | n.a. | n.a. | n.a. | n.a. |
| warming | 0.13^T^ | 0.73^T^ | 0.02^T^ | 0.88^T^ | n.a. | n.a. | n.a. | n.a. | n.a. | n.a. | n.a. | n.a. |
| fertilization | 5.92^T^ | 0.020^T^ | 4.34^T^ | 0.044^T^ | n.a. | n.a. | n.a. | n.a. | n.a. | n.a. | n.a. | n.a. |
| warm × fert | 0.22^T^ | 0.64^T^ | 0.17^T^ | 0.68^T^ | n.a. | n.a. | n.a. | n.a. | n.a. | n.a. | n.a. | n.a. |
| *11 years-old* | | | | | | | | | | | | |
| shading | 2.45 | 0.14 | 2.79 | 0.11 | n.a. | n.a. | n.a. | n.a. | n.a. | n.a. | n.a. | n.a. |
| warming | 0.02 | 0.90 | 0.12 | 0.73 | n.a. | n.a. | n.a. | n.a. | n.a. | n.a. | n.a. | n.a. |
| fertilization | 6.36 | 0.017 | 3.73 | 0.063 | n.a. | n.a. | n.a. | n.a. | n.a. | n.a. | n.a. | n.a. |
| warm × fert | 6.42 | 0.017 | 4.27 | 0.048 | n.a. | n.a. | n.a. | n.a. | n.a. | n.a. | n.a. | n.a. |
| *12 years-old* | | | | | | | | | | | | |
| shading | 1.80 | 0.21 | 2.01 | 0.19 | n.a. | n.a. | n.a. | n.a. | n.a. | n.a. | n.a. | n.a. |
| warming | 0.52^T^ | 0.48^T^ | <0.01 | 0.96 | n.a. | n.a. | n.a. | n.a. | n.a. | n.a. | n.a. | n.a. |
| fertilization | 1.86^T^ | 0.19^T^ | 3.11 | 0.095 | n.a. | n.a. | n.a. | n.a. | n.a. | n.a. | n.a. | n.a. |
| warm × fert | 1.48^T^ | 0.24^T^ | 0.10 | 0.76 | n.a. | n.a. | n.a. | n.a. | n.a. | n.a. | n.a. | n.a. |

^T^: the superscript T indicates that the data were transformed prior the ANOVA analysis; n.a.: no data available
